# Correspondence

**Published:** 1885-01

**Authors:** 


### CORRESPONDENCE.

*To the Editors of the* Journal of Comparative Medicine and Surgery:

Gentlemen—The communication in the last issue of your journal relative to the Columbia Veterinary College has been brought to my notice.

In reply I would say that from papers presented to me at the time of the transfer to the American Veterinary College of all the privileges and charter of the Columbia, including resignations, resolutions, etc., there is no doubt as to the final extinguishment of that institution, and that an attempt to re-establish the institution, or to re-invest it with corporate powers on the part of those who have pretended to do so, will, if persisted in, pave the way to serious complications, and result in discomfiture to any one attempting to continue in such a course. I shall be pleased to show you the proofs of the above statement, and hope to satisfy you of the veracity and propriety of our action in the matter.

Respectfully yours,

SAMUEL MARSH,

President American Veterinary College.

---

*To the Editors of the* JOURNAL OF COMPARATIVE MEDICINE AND SURGERY:

GENTLEMEN—Among the many institutions calculated to advance the interests of the profession of veterinary medicine, veterinary societies hold a high place. I have always considered it to be the first duty of the veterinarian to devote his attention particularly to their establishment, and I have myself attempted several times to form one, but without success. The cause of failure has been that the members wish to be all officers and no soldiers. Self-interest is their principal aim, and the result is that, after a few meetings, the affair aborts.

Now, if our brethren, in forming their associations, would elect their officers promptly and then proceed to the main business, namely, the writing and reading of such cases of interest as they meet with in their practice, and the discussion of them by the members, they would be doing a great work, not only improving their own minds, but also promoting the advancement of the profession.

Great care should be taken in accepting candidates for admission, as in no other profession is there such a number of rascals, and my opinion is that more good can be accomplished by a few good than by a large number of indifferent members.

Very truly yours,

H. E. EARL, V.S.

West New Brighton, Staten Island, N. Y.

